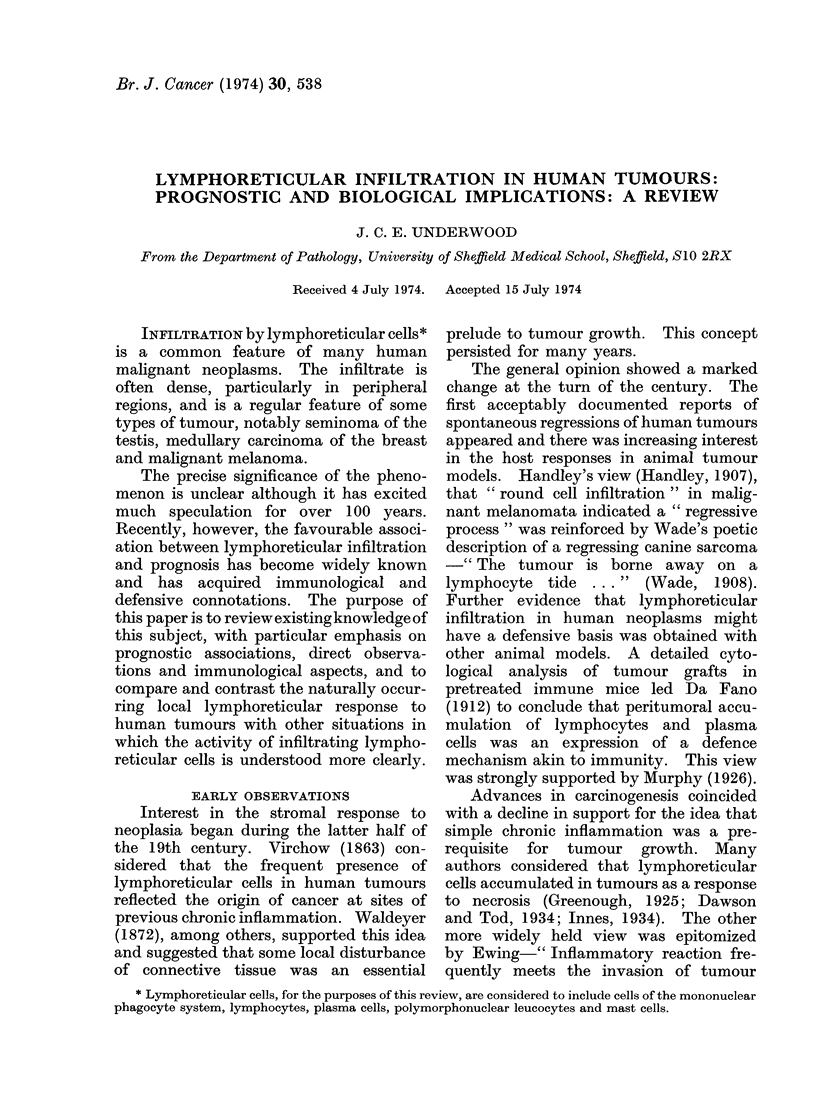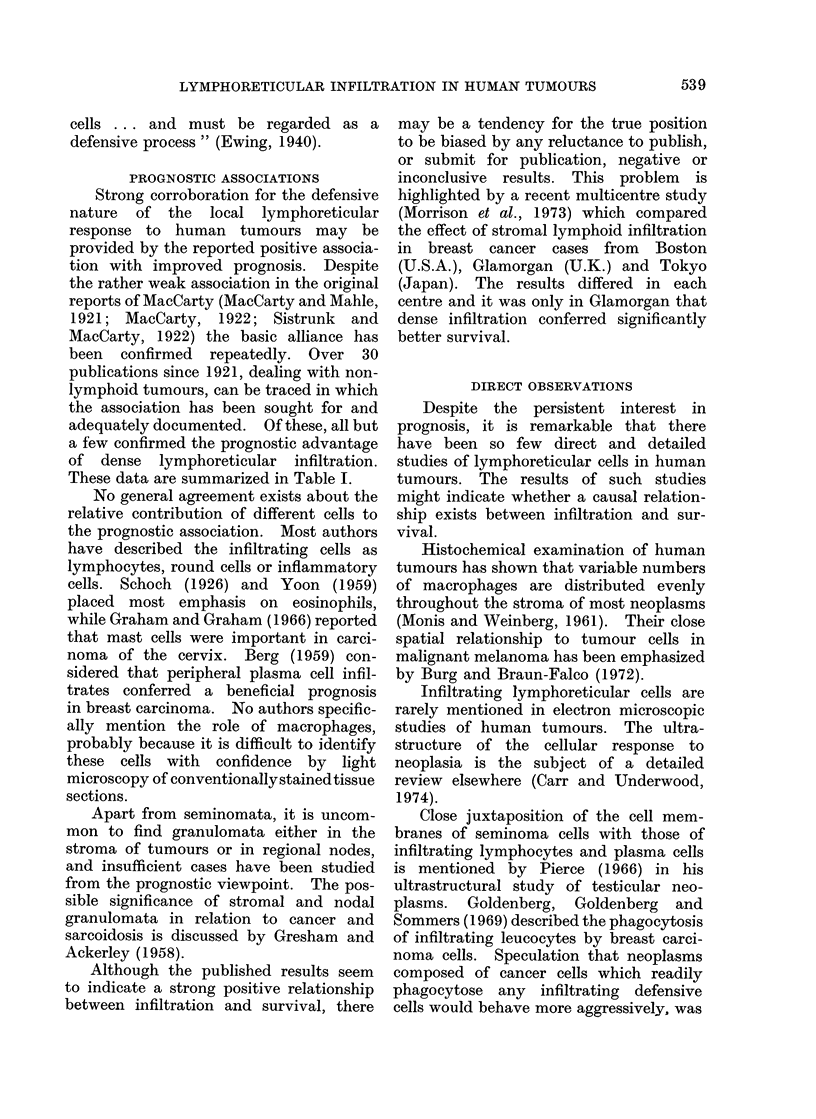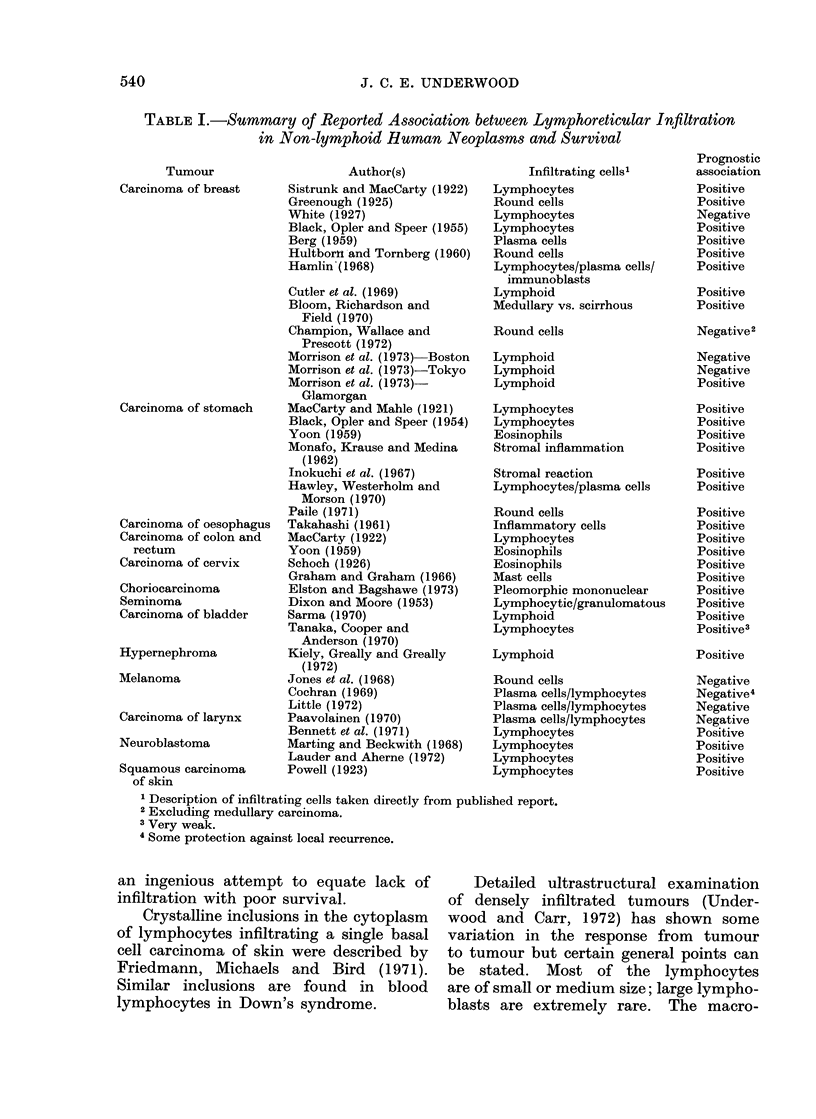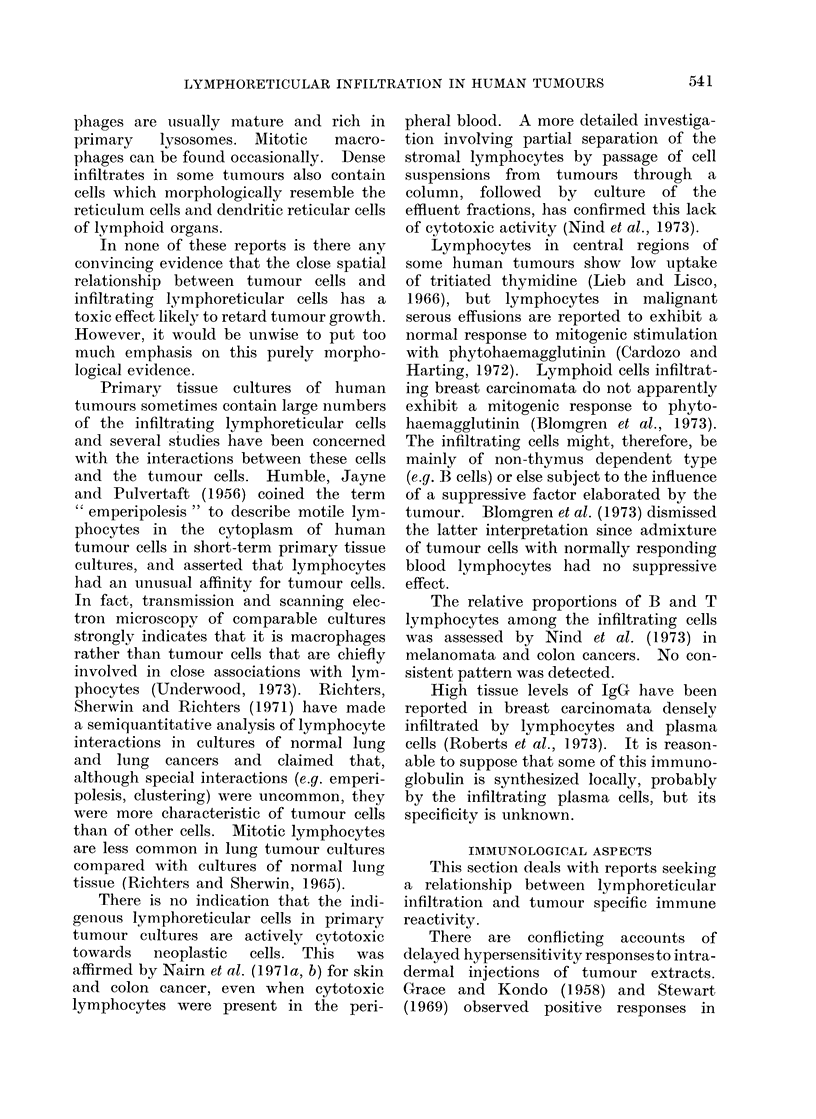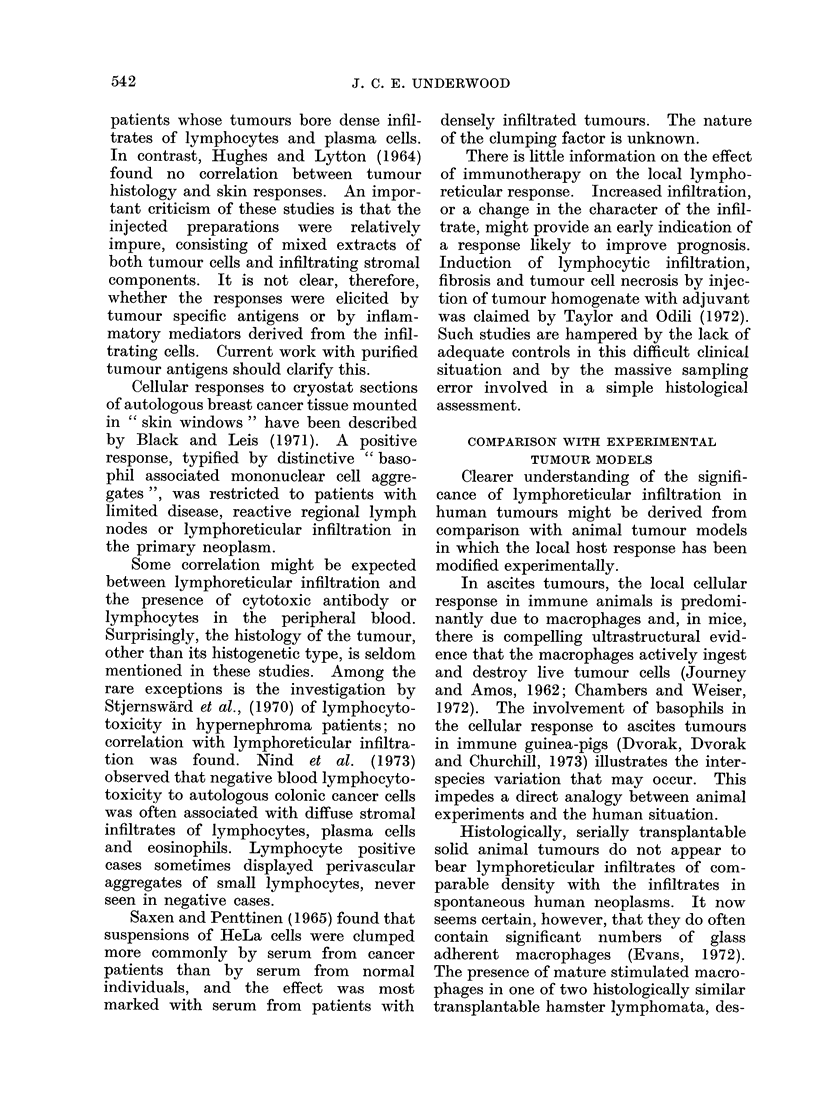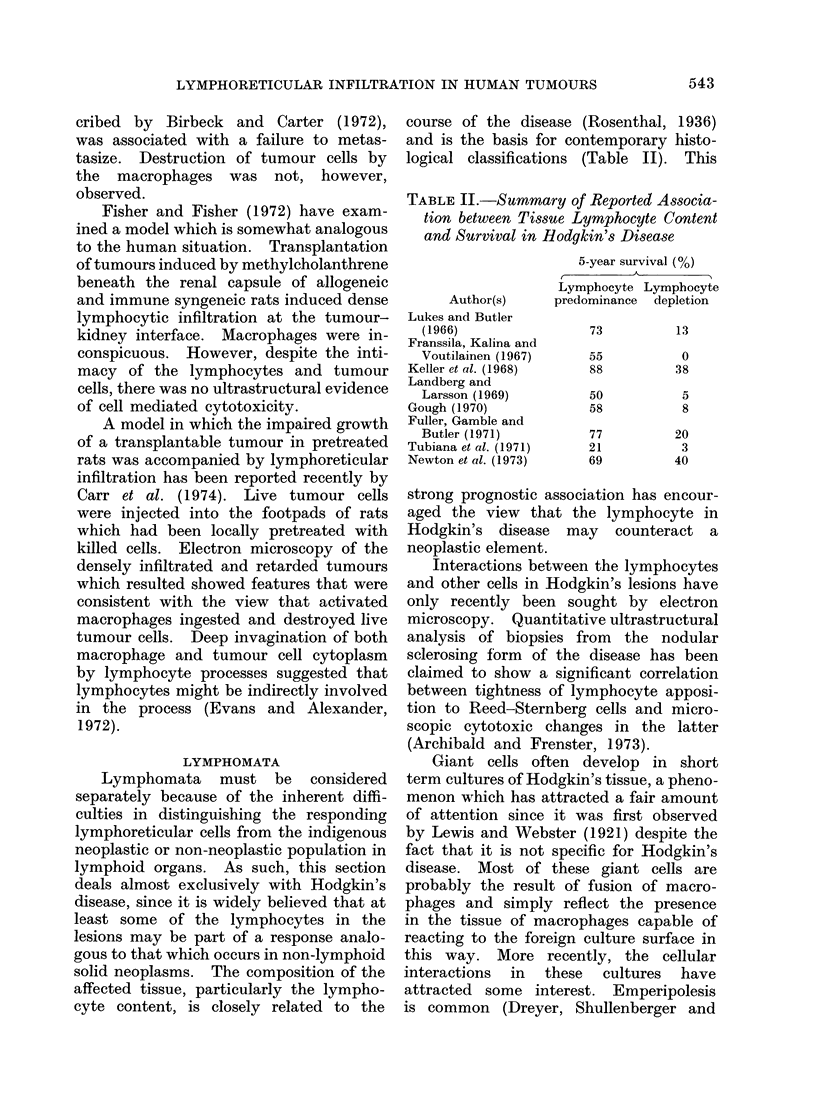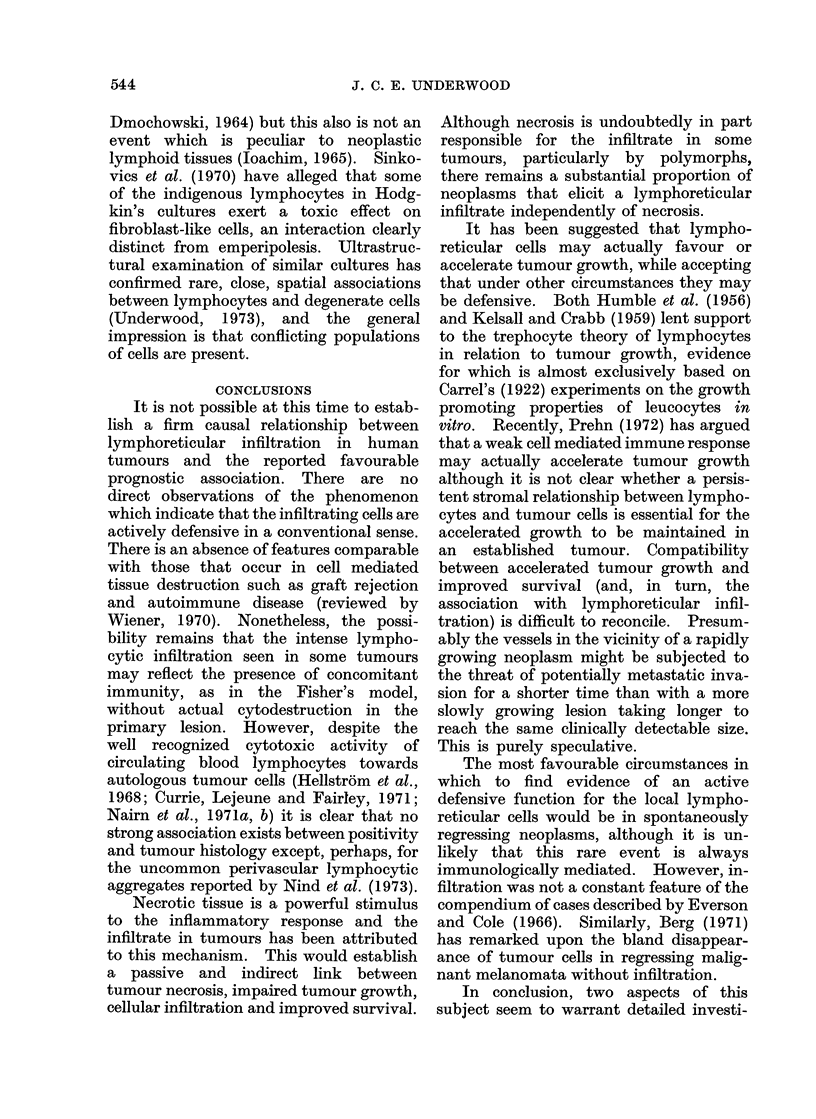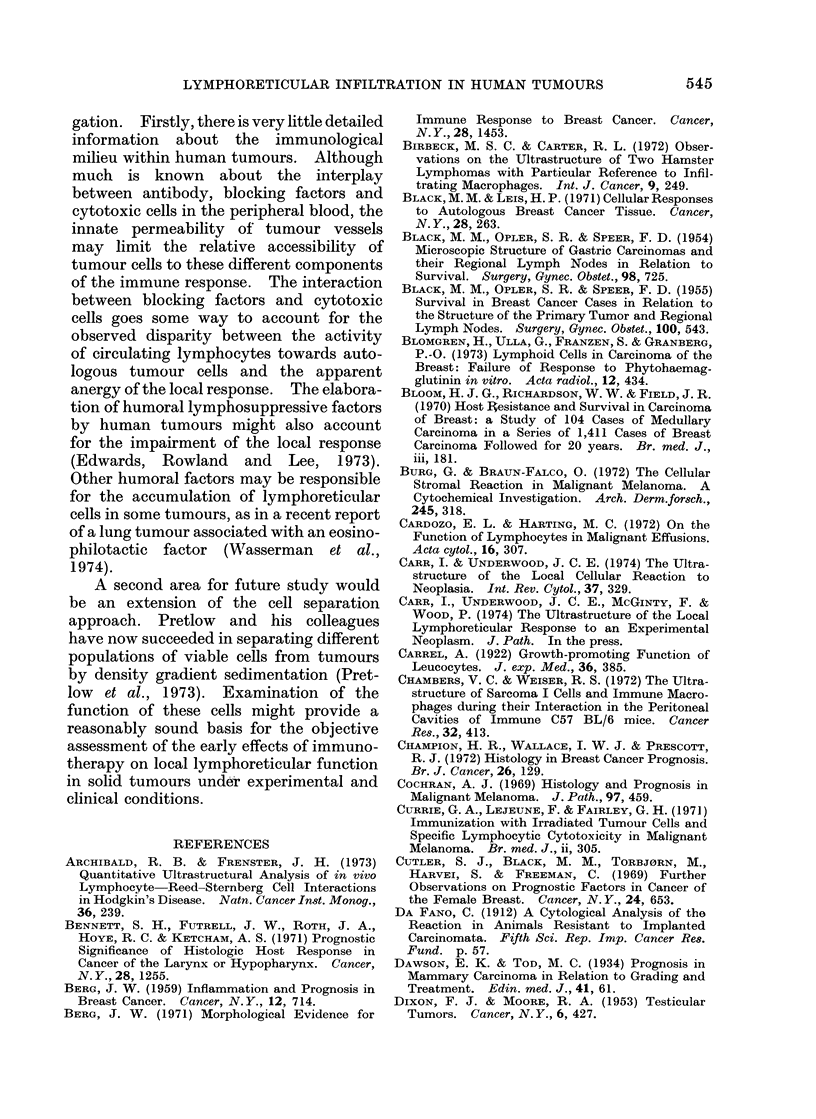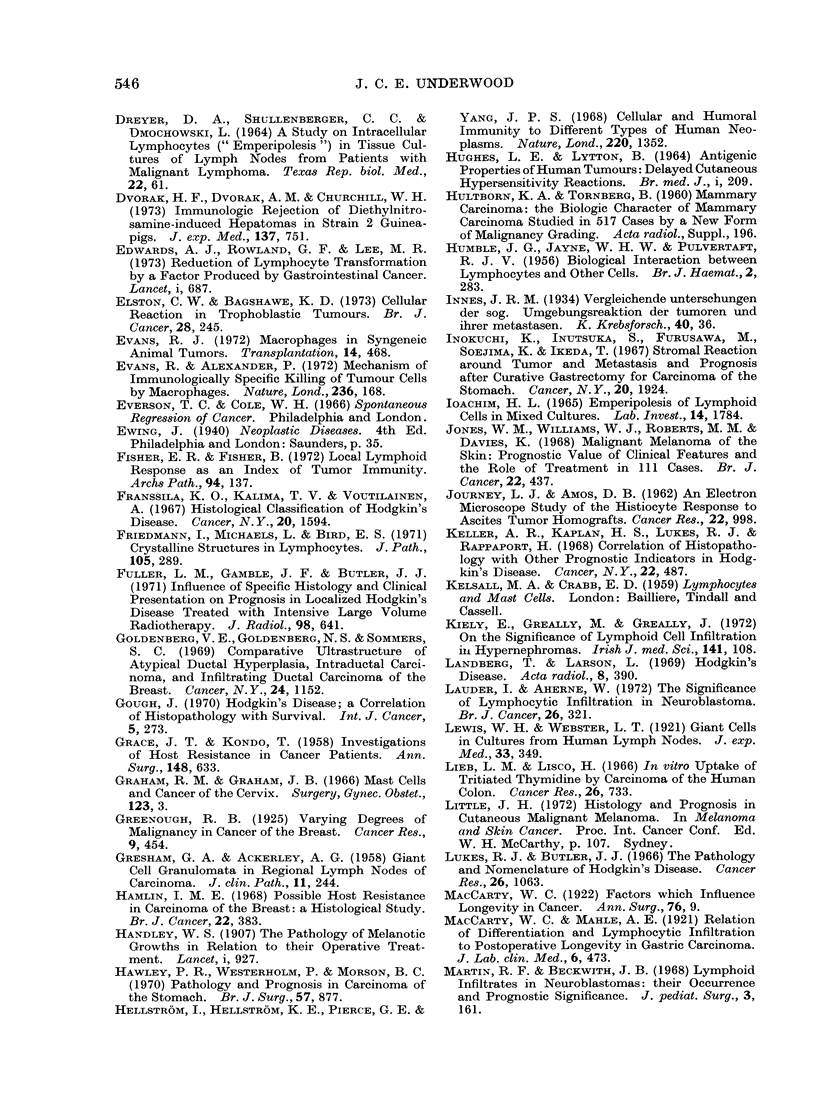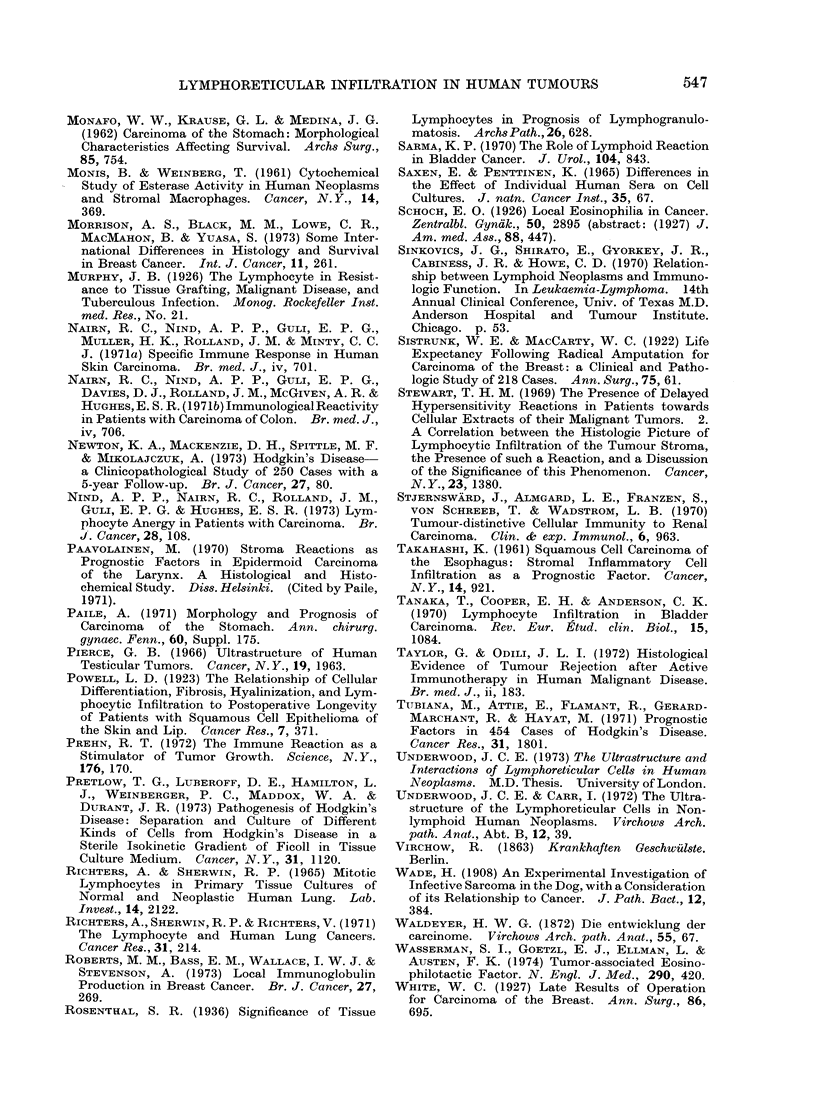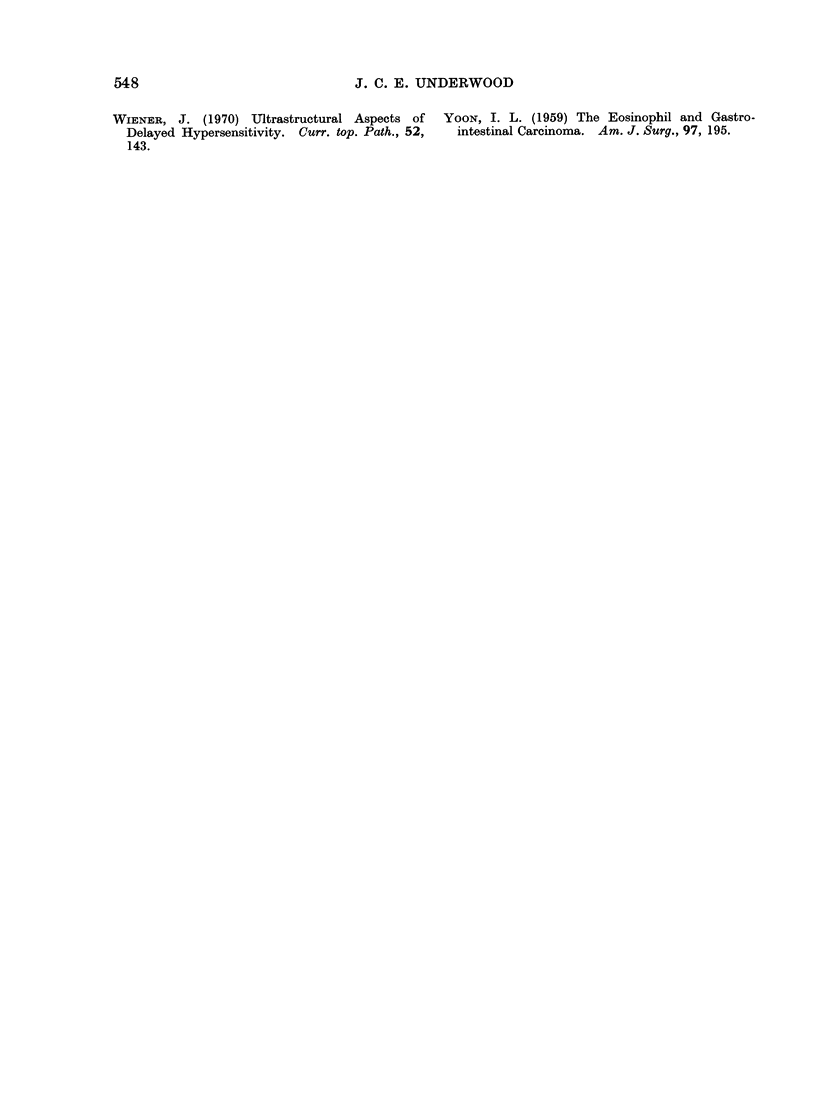# Lymphoreticular infiltration in human tumours: prognostic and biological implications: a review.

**DOI:** 10.1038/bjc.1974.233

**Published:** 1974-12

**Authors:** J. C. Underwood


*Br. J. Cancer* (1974) **30**, 538

### LYMPHORETICULAR INFILTRATION IN HUMAN TUMOURS: PROGNOSTIC AND BIOLOGICAL IMPLICATIONS: A REVIEW

J. C. E. UNDERWOOD

*From the Department of Pathology, University of Sheffield Medical School, Sheffield, S10 2RX*

Received 4 July 1974. Accepted 15 July 1974

INFILTRATION by lymphoreticular cells* is a common feature of many human malignant neoplasms. The infiltrate is often dense, particularly in peripheral regions, and is a regular feature of some types of tumour, notably seminoma of the testis, medullary carcinoma of the breast and malignant melanoma.

The precise significance of the phenomenon is unclear although it has excited much speculation for over 100 years. Recently, however, the favourable association between lymphoreticular infiltration and prognosis has become widely known and has acquired immunological and defensive connotations. The purpose of this paper is to review existing knowledge of this subject, with particular emphasis on prognostic associations, direct observations and immunological aspects, and to compare and contrast the naturally occurring local lymphoreticular response to human tumours with other situations in which the activity of infiltrating lymphoreticular cells is understood more clearly.

### EARLY OBSERVATIONS

Interest in the stromal response to neoplasia began during the latter half of the 19th century. Virchow (1863) considered that the frequent presence of lymphoreticular cells in human tumours reflected the origin of cancer at sites of previous chronic inflammation. Waldeyer (1872), among others, supported this idea and suggested that some local disturbance of connective tissue was an essential prelude to tumour growth. This concept persisted for many years.

The general opinion showed a marked change at the turn of the century. The first acceptably documented reports of spontaneous regressions of human tumours appeared and there was increasing interest in the host responses in animal tumour models. Handley's view (Handley, 1907), that " round cell infiltration " in malignant melanomata indicated a " regressive process " was reinforced by Wade's poetic description of a regressing canine sarcoma —" The tumour is borne away on a lymphocyte tide . . . " (Wade, 1908). Further evidence that lymphoreticular infiltration in human neoplasms might have a defensive basis was obtained with other animal models. A detailed cytological analysis of tumour grafts in pretreated immune mice led Da Fano (1912) to conclude that peritumoral accumulation of lymphocytes and plasma cells was an expression of a defence mechanism akin to immunity. This view was strongly supported by Murphy (1926).

Advances in carcinogenesis coincided with a decline in support for the idea that simple chronic inflammation was a prerequisite for tumour growth. Many authors considered that lymphoreticular cells accumulated in tumours as a response to necrosis (Greenough, 1925; Dawson and Tod, 1934; Innes, 1934). The other more widely held view was epitomized by Ewing—" Inflammatory reaction frequently meets the invasion of tumour

* Lymphoreticular cells, for the purposes of this review, are considered to include cells of the mononuclear phagocyte system, lymphocytes, plasma cells, polymorphonuclear leucocytes and mast cells.

cells ... and must be regarded as a defensive process " (Ewing, 1940).

### PROGNOSTIC ASSOCIATIONS

Strong corroboration for the defensive nature of the local lymphoreticular response to human tumours may be provided by the reported positive association with improved prognosis. Despite the rather weak association in the original reports of MacCarty (MacCarty and Mahle, 1921; MacCarty, 1922; Sistrunk and MacCarty, 1922) the basic alliance has been confirmed repeatedly. Over 30 publications since 1921, dealing with non-lymphoid tumours, can be traced in which the association has been sought for and adequately documented. Of these, all but a few confirmed the prognostic advantage of dense lymphoreticular infiltration. These data are summarized in Table I.

No general agreement exists about the relative contribution of different cells to the prognostic association. Most authors have described the infiltrating cells as lymphocytes, round cells or inflammatory cells. Schoch (1926) and Yoon (1959) placed most emphasis on eosinophils, while Graham and Graham (1966) reported that mast cells were important in carcinoma of the cervix. Berg (1959) considered that peripheral plasma cell infiltrates conferred a beneficial prognosis in breast carcinoma. No authors specifically mention the role of macrophages, probably because it is difficult to identify these cells with confidence by light microscopy of conventionally stained tissue sections.

Apart from seminomata, it is uncommon to find granulomata either in the stroma of tumours or in regional nodes, and insufficient cases have been studied from the prognostic viewpoint. The possible significance of stromal and nodal granulomata in relation to cancer and sarcoidosis is discussed by Gresham and Ackerley (1958).

Although the published results seem to indicate a strong positive relationship between infiltration and survival, there may be a tendency for the true position to be biased by any reluctance to publish, or submit for publication, negative or inconclusive results. This problem is highlighted by a recent multicentre study (Morrison *et al.*, 1973) which compared the effect of stromal lymphoid infiltration in breast cancer cases from Boston (U.S.A.), Glamorgan (U.K.) and Tokyo (Japan). The results differed in each centre and it was only in Glamorgan that dense infiltration conferred significantly better survival.

### DIRECT OBSERVATIONS

Despite the persistent interest in prognosis, it is remarkable that there have been so few direct and detailed studies of lymphoreticular cells in human tumours. The results of such studies might indicate whether a causal relationship exists between infiltration and survival.

Histochemical examination of human tumours has shown that variable numbers of macrophages are distributed evenly throughout the stroma of most neoplasms (Monis and Weinberg, 1961). Their close spatial relationship to tumour cells in malignant melanoma has been emphasized by Burg and Braun-Falco (1972).

Infiltrating lymphoreticular cells are rarely mentioned in electron microscopic studies of human tumours. The ultrastructure of the cellular response to neoplasia is the subject of a detailed review elsewhere (Carr and Underwood, 1974).

Close juxtaposition of the cell membranes of seminoma cells with those of infiltrating lymphocytes and plasma cells is mentioned by Pierce (1966) in his ultrastructural study of testicular neoplasms. Goldenberg, Goldenberg and Sommers (1969) described the phagocytosis of infiltrating leucocytes by breast carcinoma cells. Speculation that neoplasms composed of cancer cells which readily phagocytose any infiltrating defensive cells would behave more aggressively, was

TABLE I.—*Summary of Reported Association between Lymphoreticular Infiltration in Non-lymphoid Human Neoplasms and Survival*

| Tumour | Author(s) | Infiltrating cells[1] | Prognostic association |
|---|---|---|---|
| Carcinoma of breast | Sistrunk and MacCarty (1922) | Lymphocytes | Positive |
| | Greenough (1925) | Round cells | Positive |
| | White (1927) | Lymphocytes | Negative |
| | Black, Opler and Speer (1955) | Lymphocytes | Positive |
| | Berg (1959) | Plasma cells | Positive |
| | Hultborn and Tornberg (1960) | Round cells | Positive |
| | Hamlin (1968) | Lymphocytes/plasma cells/ immunoblasts | Positive |
| | Cutler *et al.* (1969) | Lymphoid | Positive |
| | Bloom, Richardson and Field (1970) | Medullary vs. scirrhous | Positive |
| | Champion, Wallace and Prescott (1972) | Round cells | Negative[2] |
| | Morrison *et al.* (1973)—Boston | Lymphoid | Negative |
| | Morrison *et al.* (1973)—Tokyo | Lymphoid | Negative |
| | Morrison *et al.* (1973)—Glamorgan | Lymphoid | Positive |
| Carcinoma of stomach | MacCarty and Mahle (1921) | Lymphocytes | Positive |
| | Black, Opler and Speer (1954) | Lymphocytes | Positive |
| | Yoon (1959) | Eosinophils | Positive |
| | Monafo, Krause and Medina (1962) | Stromal inflammation | Positive |
| | Inokuchi *et al.* (1967) | Stromal reaction | Positive |
| | Hawley, Westerholm and Morson (1970) | Lymphocytes/plasma cells | Positive |
| | Paile (1971) | Round cells | Positive |
| Carcinoma of oesophagus | Takahashi (1961) | Inflammatory cells | Positive |
| Carcinoma of colon and rectum | MacCarty (1922) | Lymphocytes | Positive |
| | Yoon (1959) | Eosinophils | Positive |
| Carcinoma of cervix | Schoch (1926) | Eosinophils | Positive |
| | Graham and Graham (1966) | Mast cells | Positive |
| Choriocarcinoma | Elston and Bagshawe (1973) | Pleomorphic mononuclear | Positive |
| Seminoma | Dixon and Moore (1953) | Lymphocytic/granulomatous | Positive |
| Carcinoma of bladder | Sarma (1970) | Lymphoid | Positive |
| | Tanaka, Cooper and Anderson (1970) | Lymphocytes | Positive[3] |
| Hypernephroma | Kiely, Greally and Greally (1972) | Lymphoid | Positive |
| Melanoma | Jones *et al.* (1968) | Round cells | Negative |
| | Cochran (1969) | Plasma cells/lymphocytes | Negative[4] |
| | Little (1972) | Plasma cells/lymphocytes | Negative |
| Carcinoma of larynx | Paavolainen (1970) | Plasma cells/lymphocytes | Negative |
| | Bennett *et al.* (1971) | Lymphocytes | Positive |
| Neuroblastoma | Marting and Beckwith (1968) | Lymphocytes | Positive |
| | Lauder and Aherne (1972) | Lymphocytes | Positive |
| Squamous carcinoma of skin | Powell (1923) | Lymphocytes | Positive |

[1] Description of infiltrating cells taken directly from published report.
[2] Excluding medullary carcinoma.
[3] Very weak.
[4] Some protection against local recurrence.

an ingenious attempt to equate lack of infiltration with poor survival.

Crystalline inclusions in the cytoplasm of lymphocytes infiltrating a single basal cell carcinoma of skin were described by Friedmann, Michaels and Bird (1971). Similar inclusions are found in blood lymphocytes in Down's syndrome.

Detailed ultrastructural examination of densely infiltrated tumours (Underwood and Carr, 1972) has shown some variation in the response from tumour to tumour but certain general points can be stated. Most of the lymphocytes are of small or medium size; large lymphoblasts are extremely rare. The macrophages are usually mature and rich in primary lysosomes. Mitotic macrophages can be found occasionally. Dense infiltrates in some tumours also contain cells which morphologically resemble the reticulum cells and dendritic reticular cells of lymphoid organs.

In none of these reports is there any convincing evidence that the close spatial relationship between tumour cells and infiltrating lymphoreticular cells has a toxic effect likely to retard tumour growth. However, it would be unwise to put too much emphasis on this purely morphological evidence.

Primary tissue cultures of human tumours sometimes contain large numbers of the infiltrating lymphoreticular cells and several studies have been concerned with the interactions between these cells and the tumour cells. Humble, Jayne and Pulvertaft (1956) coined the term " emperipolesis " to describe motile lymphocytes in the cytoplasm of human tumour cells in short-term primary tissue cultures, and asserted that lymphocytes had an unusual affinity for tumour cells. In fact, transmission and scanning electron microscopy of comparable cultures strongly indicates that it is macrophages rather than tumour cells that are chiefly involved in close associations with lymphocytes (Underwood, 1973). Richters, Sherwin and Richters (1971) have made a semiquantitative analysis of lymphocyte interactions in cultures of normal lung and lung cancers and claimed that, although special interactions (*e.g.* emperipolesis, clustering) were uncommon, they were more characteristic of tumour cells than of other cells. Mitotic lymphocytes are less common in lung tumour cultures compared with cultures of normal lung tissue (Richters and Sherwin, 1965).

There is no indication that the indigenous lymphoreticular cells in primary tumour cultures are actively cytotoxic towards neoplastic cells. This was affirmed by Nairn *et al.* (1971*a, b*) for skin and colon cancer, even when cytotoxic lymphocytes were present in the peripheral blood. A more detailed investigation involving partial separation of the stromal lymphocytes by passage of cell suspensions from tumours through a column, followed by culture of the effluent fractions, has confirmed this lack of cytotoxic activity (Nind *et al.*, 1973).

Lymphocytes in central regions of some human tumours show low uptake of tritiated thymidine (Lieb and Lisco, 1966), but lymphocytes in malignant serous effusions are reported to exhibit a normal response to mitogenic stimulation with phytohaemagglutinin (Cardozo and Harting, 1972). Lymphoid cells infiltrating breast carcinomata do not apparently exhibit a mitogenic response to phytohaemagglutinin (Blomgren *et al.*, 1973). The infiltrating cells might, therefore, be mainly of non-thymus dependent type (*e.g.* B cells) or else subject to the influence of a suppressive factor elaborated by the tumour. Blomgren *et al.* (1973) dismissed the latter interpretation since admixture of tumour cells with normally responding blood lymphocytes had no suppressive effect.

The relative proportions of B and T lymphocytes among the infiltrating cells was assessed by Nind *et al.* (1973) in melanomata and colon cancers. No consistent pattern was detected.

High tissue levels of IgG have been reported in breast carcinomata densely infiltrated by lymphocytes and plasma cells (Roberts *et al.*, 1973). It is reasonable to suppose that some of this immunoglobulin is synthesized locally, probably by the infiltrating plasma cells, but its specificity is unknown.

### IMMUNOLOGICAL ASPECTS

This section deals with reports seeking a relationship between lymphoreticular infiltration and tumour specific immune reactivity.

There are conflicting accounts of delayed hypersensitivity responses to intradermal injections of tumour extracts. Grace and Kondo (1958) and Stewart (1969) observed positive responses in patients whose tumours bore dense infiltrates of lymphocytes and plasma cells. In contrast, Hughes and Lytton (1964) found no correlation between tumour histology and skin responses. An important criticism of these studies is that the injected preparations were relatively impure, consisting of mixed extracts of both tumour cells and infiltrating stromal components. It is not clear, therefore, whether the responses were elicited by tumour specific antigens or by inflammatory mediators derived from the infiltrating cells. Current work with purified tumour antigens should clarify this.

Cellular responses to cryostat sections of autologous breast cancer tissue mounted in " skin windows " have been described by Black and Leis (1971). A positive response, typified by distinctive " basophil associated mononuclear cell aggregates ", was restricted to patients with limited disease, reactive regional lymph nodes or lymphoreticular infiltration in the primary neoplasm.

Some correlation might be expected between lymphoreticular infiltration and the presence of cytotoxic antibody or lymphocytes in the peripheral blood. Surprisingly, the histology of the tumour, other than its histogenetic type, is seldom mentioned in these studies. Among the rare exceptions is the investigation by Stjernswärd *et al.*, (1970) of lymphocytotoxicity in hypernephroma patients; no correlation with lymphoreticular infiltration was found. Nind *et al.* (1973) observed that negative blood lymphocytotoxicity to autologous colonic cancer cells was often associated with diffuse stromal infiltrates of lymphocytes, plasma cells and eosinophils. Lymphocyte positive cases sometimes displayed perivascular aggregates of small lymphocytes, never seen in negative cases.

Saxen and Penttinen (1965) found that suspensions of HeLa cells were clumped more commonly by serum from cancer patients than by serum from normal individuals, and the effect was most marked with serum from patients with densely infiltrated tumours. The nature of the clumping factor is unknown.

There is little information on the effect of immunotherapy on the local lymphoreticular response. Increased infiltration, or a change in the character of the infiltrate, might provide an early indication of a response likely to improve prognosis. Induction of lymphocytic infiltration, fibrosis and tumour cell necrosis by injection of tumour homogenate with adjuvant was claimed by Taylor and Odili (1972). Such studies are hampered by the lack of adequate controls in this difficult clinical situation and by the massive sampling error involved in a simple histological assessment.

### COMPARISON WITH EXPERIMENTAL TUMOUR MODELS

Clearer understanding of the significance of lymphoreticular infiltration in human tumours might be derived from comparison with animal tumour models in which the local host response has been modified experimentally.

In ascites tumours, the local cellular response in immune animals is predominantly due to macrophages and, in mice, there is compelling ultrastructural evidence that the macrophages actively ingest and destroy live tumour cells (Journey and Amos, 1962; Chambers and Weiser, 1972). The involvement of basophils in the cellular response to ascites tumours in immune guinea-pigs (Dvorak, Dvorak and Churchill, 1973) illustrates the interspecies variation that may occur. This impedes a direct analogy between animal experiments and the human situation.

Histologically, serially transplantable solid animal tumours do not appear to bear lymphoreticular infiltrates of comparable density with the infiltrates in spontaneous human neoplasms. It now seems certain, however, that they do often contain significant numbers of glass adherent macrophages (Evans, 1972). The presence of mature stimulated macrophages in one of two histologically similar transplantable hamster lymphomata, described by Birbeck and Carter (1972), was associated with a failure to metastasize. Destruction of tumour cells by the macrophages was not, however, observed.

Fisher and Fisher (1972) have examined a model which is somewhat analogous to the human situation. Transplantation of tumours induced by methylcholanthrene beneath the renal capsule of allogeneic and immune syngeneic rats induced dense lymphocytic infiltration at the tumour–kidney interface. Macrophages were inconspicuous. However, despite the intimacy of the lymphocytes and tumour cells, there was no ultrastructural evidence of cell mediated cytotoxicity.

A model in which the impaired growth of a transplantable tumour in pretreated rats was accompanied by lymphoreticular infiltration has been reported recently by Carr *et al.* (1974). Live tumour cells were injected into the footpads of rats which had been locally pretreated with killed cells. Electron microscopy of the densely infiltrated and retarded tumours which resulted showed features that were consistent with the view that activated macrophages ingested and destroyed live tumour cells. Deep invagination of both macrophage and tumour cell cytoplasm by lymphocyte processes suggested that lymphocytes might be indirectly involved in the process (Evans and Alexander, 1972).

### LYMPHOMATA

Lymphomata must be considered separately because of the inherent difficulties in distinguishing the responding lymphoreticular cells from the indigenous neoplastic or non-neoplastic population in lymphoid organs. As such, this section deals almost exclusively with Hodgkin's disease, since it is widely believed that at least some of the lymphocytes in the lesions may be part of a response analogous to that which occurs in non-lymphoid solid neoplasms. The composition of the affected tissue, particularly the lymphocyte content, is closely related to the course of the disease (Rosenthal, 1936) and is the basis for contemporary histological classifications (Table II). This

TABLE II.—*Summary of Reported Association between Tissue Lymphocyte Content and Survival in Hodgkin's Disease*

| Author(s) | 5-year survival (%) | |
|---|---|---|
| | Lymphocyte predominance | Lymphocyte depletion |
| Lukes and Butler (1966) | 73 | 13 |
| Franssila, Kalina and Voutilainen (1967) | 55 | 0 |
| Keller *et al.* (1968) | 88 | 38 |
| Landberg and Larsson (1969) | 50 | 5 |
| Gough (1970) | 58 | 8 |
| Fuller, Gamble and Butler (1971) | 77 | 20 |
| Tubiana *et al.* (1971) | 21 | 3 |
| Newton *et al.* (1973) | 69 | 40 |

strong prognostic association has encouraged the view that the lymphocyte in Hodgkin's disease may counteract a neoplastic element.

Interactions between the lymphocytes and other cells in Hodgkin's lesions have only recently been sought by electron microscopy. Quantitative ultrastructural analysis of biopsies from the nodular sclerosing form of the disease has been claimed to show a significant correlation between tightness of lymphocyte apposition to Reed–Sternberg cells and microscopic cytotoxic changes in the latter (Archibald and Frenster, 1973).

Giant cells often develop in short term cultures of Hodgkin's tissue, a phenomenon which has attracted a fair amount of attention since it was first observed by Lewis and Webster (1921) despite the fact that it is not specific for Hodgkin's disease. Most of these giant cells are probably the result of fusion of macrophages and simply reflect the presence in the tissue of macrophages capable of reacting to the foreign culture surface in this way. More recently, the cellular interactions in these cultures have attracted some interest. Emperipolesis is common (Dreyer, Shullenberger and

Dmochowski, 1964) but this also is not an event which is peculiar to neoplastic lymphoid tissues (Ioachim, 1965). Sinkovics *et al.* (1970) have alleged that some of the indigenous lymphocytes in Hodgkin's cultures exert a toxic effect on fibroblast-like cells, an interaction clearly distinct from emperipolesis. Ultrastructural examination of similar cultures has confirmed rare, close, spatial associations between lymphocytes and degenerate cells (Underwood, 1973), and the general impression is that conflicting populations of cells are present.

### CONCLUSIONS

It is not possible at this time to establish a firm causal relationship between lymphoreticular infiltration in human tumours and the reported favourable prognostic association. There are no direct observations of the phenomenon which indicate that the infiltrating cells are actively defensive in a conventional sense. There is an absence of features comparable with those that occur in cell mediated tissue destruction such as graft rejection and autoimmune disease (reviewed by Wiener, 1970). Nonetheless, the possibility remains that the intense lymphocytic infiltration seen in some tumours may reflect the presence of concomitant immunity, as in the Fisher's model, without actual cytodestruction in the primary lesion. However, despite the well recognized cytotoxic activity of circulating blood lymphocytes towards autologous tumour cells (Hellström *et al.*, 1968; Currie, Lejeune and Fairley, 1971; Nairn *et al.*, 1971*a*, *b*) it is clear that no strong association exists between positivity and tumour histology except, perhaps, for the uncommon perivascular lymphocytic aggregates reported by Nind *et al.* (1973).

Necrotic tissue is a powerful stimulus to the inflammatory response and the infiltrate in tumours has been attributed to this mechanism. This would establish a passive and indirect link between tumour necrosis, impaired tumour growth, cellular infiltration and improved survival. Although necrosis is undoubtedly in part responsible for the infiltrate in some tumours, particularly by polymorphs, there remains a substantial proportion of neoplasms that elicit a lymphoreticular infiltrate independently of necrosis.

It has been suggested that lymphoreticular cells may actually favour or accelerate tumour growth, while accepting that under other circumstances they may be defensive. Both Humble *et al.* (1956) and Kelsall and Crabb (1959) lent support to the trephocyte theory of lymphocytes in relation to tumour growth, evidence for which is almost exclusively based on Carrel's (1922) experiments on the growth promoting properties of leucocytes *in vitro*. Recently, Prehn (1972) has argued that a weak cell mediated immune response may actually accelerate tumour growth although it is not clear whether a persistent stromal relationship between lymphocytes and tumour cells is essential for the accelerated growth to be maintained in an established tumour. Compatibility between accelerated tumour growth and improved survival (and, in turn, the association with lymphoreticular infiltration) is difficult to reconcile. Presumably the vessels in the vicinity of a rapidly growing neoplasm might be subjected to the threat of potentially metastatic invasion for a shorter time than with a more slowly growing lesion taking longer to reach the same clinically detectable size. This is purely speculative.

The most favourable circumstances in which to find evidence of an active defensive function for the local lymphoreticular cells would be in spontaneously regressing neoplasms, although it is unlikely that this rare event is always immunologically mediated. However, infiltration was not a constant feature of the compendium of cases described by Everson and Cole (1966). Similarly, Berg (1971) has remarked upon the bland disappearance of tumour cells in regressing malignant melanomata without infiltration.

In conclusion, two aspects of this subject seem to warrant detailed investigation. Firstly, there is very little detailed information about the immunological milieu within human tumours. Although much is known about the interplay between antibody, blocking factors and cytotoxic cells in the peripheral blood, the innate permeability of tumour vessels may limit the relative accessibility of tumour cells to these different components of the immune response. The interaction between blocking factors and cytotoxic cells goes some way to account for the observed disparity between the activity of circulating lymphocytes towards autologous tumour cells and the apparent anergy of the local response. The elaboration of humoral lymphosuppressive factors by human tumours might also account for the impairment of the local response (Edwards, Rowland and Lee, 1973). Other humoral factors may be responsible for the accumulation of lymphoreticular cells in some tumours, as in a recent report of a lung tumour associated with an eosinophilotactic factor (Wasserman *et al.*, 1974).

A second area for future study would be an extension of the cell separation approach. Pretlow and his colleagues have now succeeded in separating different populations of viable cells from tumours by density gradient sedimentation (Pretlow *et al.*, 1973). Examination of the function of these cells might provide a reasonably sound basis for the objective assessment of the early effects of immunotherapy on local lymphoreticular function in solid tumours under experimental and clinical conditions.

### REFERENCES

ARCHIBALD, R. B. & FRENSTER, J. H. (1973) Quantitative Ultrastructural Analysis of *in vivo* Lymphocyte—Reed–Sternberg Cell Interactions in Hodgkin's Disease. *Natn. Cancer Inst. Monog.*, **36**, 239.

BENNETT, S. H., FUTRELL, J. W., ROTH, J. A., HOYE, R. C. & KETCHAM, A. S. (1971) Prognostic Significance of Histologic Host Response in Cancer of the Larynx or Hypopharynx. *Cancer, N.Y.*, **28**, 1255.

BERG, J. W. (1959) Inflammation and Prognosis in Breast Cancer. *Cancer, N.Y.*, **12**, 714.

BERG, J. W. (1971) Morphological Evidence for Immune Response to Breast Cancer. *Cancer, N.Y.*, **28**, 1453.

BIRBECK, M. S. C. & CARTER, R. L. (1972) Observations on the Ultrastructure of Two Hamster Lymphomas with Particular Reference to Infiltrating Macrophages. *Int. J. Cancer*, **9**, 249.

BLACK, M. M. & LEIS, H. P. (1971) Cellular Responses to Autologous Breast Cancer Tissue. *Cancer, N.Y.*, **28**, 263.

BLACK, M. M., OPLER, S. R. & SPEER, F. D. (1954) Microscopic Structure of Gastric Carcinomas and their Regional Lymph Nodes in Relation to Survival. *Surgery, Gynec. Obstet.*, **98**, 725.

BLACK, M. M., OPLER, S. R. & SPEER, F. D. (1955) Survival in Breast Cancer Cases in Relation to the Structure of the Primary Tumor and Regional Lymph Nodes. *Surgery, Gynec. Obstet.*, **100**, 543.

BLOMGREN, H., ULLA, G., FRANZEN, S. & GRANBERG, P.-O. (1973) Lymphoid Cells in Carcinoma of the Breast: Failure of Response to Phytohaemagglutinin *in vitro*. *Acta radiol.*, **12**, 434.

BLOOM, H. J. G., RICHARDSON, W. W. & FIELD, J. R. (1970) Host Resistance and Survival in Carcinoma of Breast: a Study of 104 Cases of Medullary Carcinoma in a Series of 1,411 Cases of Breast Carcinoma Followed for 20 years. *Br. med. J.*, iii, 181.

BURG, G. & BRAUN-FALCO, O. (1972) The Cellular Stromal Reaction in Malignant Melanoma. A Cytochemical Investigation. *Arch. Derm.forsch.*, **245**, 318.

CARDOZO, E. L. & HARTING, M. C. (1972) On the Function of Lymphocytes in Malignant Effusions. *Acta cytol.*, **16**, 307.

CARR, I. & UNDERWOOD, J. C. E. (1974) The Ultrastructure of the Local Cellular Reaction to Neoplasia. *Int. Rev. Cytol.*, **37**, 329.

CARR, I., UNDERWOOD, J. C. E., MCGINTY, F. & WOOD, P. (1974) The Ultrastructure of the Local Lymphoreticular Response to an Experimental Neoplasm. *J. Path.* In the press.

CARREL, A. (1922) Growth-promoting Function of Leucocytes. *J. exp. Med.*, **36**, 385.

CHAMBERS, V. C. & WEISER, R. S. (1972) The Ultrastructure of Sarcoma I Cells and Immune Macrophages during their Interaction in the Peritoneal Cavities of Immune C57 BL/6 mice. *Cancer Res.*, **32**, 413.

CHAMPION, H. R., WALLACE, I. W. J. & PRESCOTT, R. J. (1972) Histology in Breast Cancer Prognosis. *Br. J. Cancer*, **26**, 129.

COCHRAN, A. J. (1969) Histology and Prognosis in Malignant Melanoma. *J. Path.*, **97**, 459.

CURRIE, G. A., LEJEUNE, F. & FAIRLEY, G. H. (1971) Immunization with Irradiated Tumour Cells and Specific Lymphocytic Cytotoxicity in Malignant Melanoma. *Br. med. J.*, ii, 305.

CUTLER, S. J., BLACK, M. M., TORBJØRN, M., HARVEI, S. & FREEMAN, C. (1969) Further Observations on Prognostic Factors in Cancer of the Female Breast. *Cancer, N.Y.*, **24**, 653.

DA FANO, C. (1912) A Cytological Analysis of the Reaction in Animals Resistant to Implanted Carcinomata. *Fifth Sci. Rep. Imp. Cancer Res. Fund.* p. 57.

DAWSON, E. K. & TOD, M. C. (1934) Prognosis in Mammary Carcinoma in Relation to Grading and Treatment. *Edin. med. J.*, **41**, 61.

DIXON, F. J. & MOORE, R. A. (1953) Testicular Tumors. *Cancer, N.Y.*, **6**, 427.

DREYER, D. A., SHULLENBERGER, C. C. & DMOCHOWSKI, L. (1964) A Study on Intracellular Lymphocytes (" Emperipolesis ") in Tissue Cultures of Lymph Nodes from Patients with Malignant Lymphoma. *Texas Rep. biol. Med.*, **22**, 61.

DVORAK, H. F., DVORAK, A. M. & CHURCHILL, W. H. (1973) Immunologic Rejection of Diethylnitrosamine-induced Hepatomas in Strain 2 Guinea-pigs. *J. exp. Med.*, **137**, 751.

EDWARDS, A. J., ROWLAND, G. F. & LEE, M. R. (1973) Reduction of Lymphocyte Transformation by a Factor Produced by Gastrointestinal Cancer. *Lancet*, i, 687.

ELSTON, C. W. & BAGSHAWE, K. D. (1973) Cellular Reaction in Trophoblastic Tumours. *Br. J. Cancer*, **28**, 245.

EVANS, R. J. (1972) Macrophages in Syngeneic Animal Tumors. *Transplantation*, **14**, 468.

EVANS, R. & ALEXANDER, P. (1972) Mechanism of Immunologically Specific Killing of Tumour Cells by Macrophages. *Nature, Lond.*, **236**, 168.

EVERSON, T. C. & COLE, W. H. (1966) *Spontaneous Regression of Cancer*. Philadelphia and London.

EWING, J. (1940) *Neoplastic Diseases*. 4th Ed. Philadelphia and London: Saunders, p. 35.

FISHER, E. R. & FISHER, B. (1972) Local Lymphoid Response as an Index of Tumor Immunity. *Archs Path.*, **94**, 137.

FRANSSILA, K. O., KALIMA, T. V. & VOUTILAINEN, A. (1967) Histological Classification of Hodgkin's Disease. *Cancer, N.Y.*, **20**, 1594.

FRIEDMANN, I., MICHAELS, L. & BIRD, E. S. (1971) Crystalline Structures in Lymphocytes. *J. Path.*, **105**, 289.

FULLER, L. M., GAMBLE, J. F. & BUTLER, J. J. (1971) Influence of Specific Histology and Clinical Presentation on Prognosis in Localized Hodgkin's Disease Treated with Intensive Large Volume Radiotherapy. *J. Radiol.*, **98**, 641.

GOLDENBERG, V. E., GOLDENBERG, N. S. & SOMMERS, S. C. (1969) Comparative Ultrastructure of Atypical Ductal Hyperplasia, Intraductal Carcinoma, and Infiltrating Ductal Carcinoma of the Breast. *Cancer, N.Y.*, **24**, 1152.

GOUGH, J. (1970) Hodgkin's Disease; a Correlation of Histopathology with Survival. *Int. J. Cancer*, **5**, 273.

GRACE, J. T. & KONDO, T. (1958) Investigations of Host Resistance in Cancer Patients. *Ann. Surg.*, **148**, 633.

GRAHAM, R. M. & GRAHAM, J. B. (1966) Mast Cells and Cancer of the Cervix. *Surgery, Gynec. Obstet.*, **123**, 3.

GREENOUGH, R. B. (1925) Varying Degrees of Malignancy in Cancer of the Breast. *Cancer Res.*, **9**, 454.

GRESHAM, G. A. & ACKERLEY, A. G. (1958) Giant Cell Granulomata in Regional Lymph Nodes of Carcinoma. *J. clin. Path.*, **11**, 244.

HAMLIN, I. M. E. (1968) Possible Host Resistance in Carcinoma of the Breast: a Histological Study. *Br. J. Cancer*, **22**, 383.

HANDLEY, W. S. (1907) The Pathology of Melanotic Growths in Relation to their Operative Treatment. *Lancet*, i, 927.

HAWLEY, P. R., WESTERHOLM, P. & MORSON, B. C. (1970) Pathology and Prognosis in Carcinoma of the Stomach. *Br. J. Surg.*, **57**, 877.

HELLSTRÖM, I., HELLSTRÖM, K. E., PIERCE, G. E. & YANG, J. P. S. (1968) Cellular and Humoral Immunity to Different Types of Human Neoplasms. *Nature, Lond.*, **220**, 1352.

HUGHES, L. E. & LYTTON, B. (1964) Antigenic Properties of Human Tumours: Delayed Cutaneous Hypersensitivity Reactions. *Br. med. J.*, i, 209.

HULTBORN, K. A. & TORNBERG, B. (1960) Mammary Carcinoma: the Biologic Character of Mammary Carcinoma Studied in 517 Cases by a New Form of Malignancy Grading. *Acta radiol.*, Suppl., 196.

HUMBLE, J. G., JAYNE, W. H. W. & PULVERTAFT, R. J. V. (1956) Biological Interaction between Lymphocytes and Other Cells. *Br. J. Haemat.*, **2**, 283.

INNES, J. R. M. (1934) Vergleichende unterschungen der sog. Umgebungsreaktion der tumoren und ihrer metastasen. *K. Krebsforsch.*, **40**, 36.

INOKUCHI, K., INUTSUKA, S., FURUSAWA, M., SOEJIMA, K. & IKEDA, T. (1967) Stromal Reaction around Tumor and Metastasis and Prognosis after Curative Gastrectomy for Carcinoma of the Stomach. *Cancer, N.Y.*, **20**, 1924.

IOACHIM, H. L. (1965) Emperipolesis of Lymphoid Cells in Mixed Cultures. *Lab. Invest.*, **14**, 1784.

JONES, W. M., WILLIAMS, W. J., ROBERTS, M. M. & DAVIES, K. (1968) Malignant Melanoma of the Skin: Prognostic Value of Clinical Features and the Role of Treatment in 111 Cases. *Br. J. Cancer*, **22**, 437.

JOURNEY, L. J. & AMOS, D. B. (1962) An Electron Microscope Study of the Histiocyte Response to Ascites Tumor Homografts. *Cancer Res.*, **22**, 998.

KELLER, A. R., KAPLAN, H. S., LUKES, R. J. & RAPPAPORT, H. (1968) Correlation of Histopathology with Other Prognostic Indicators in Hodgkin's Disease. *Cancer, N.Y.*, **22**, 487.

KELSALL, M. A. & CRABB, E. D. (1959) *Lymphocytes and Mast Cells*. London: Bailliere, Tindall and Cassell.

KIELY, E., GREALLY, M. & GREALLY, J. (1972) On the Significance of Lymphoid Cell Infiltration in Hypernephromas. *Irish J. med. Sci.*, **141**, 108.

LANDBERG, T. & LARSON, L. (1969) Hodgkin's Disease. *Acta radiol.*, **8**, 390.

LAUDER, I. & AHERNE, W. (1972) The Significance of Lymphocytic Infiltration in Neuroblastoma. *Br. J. Cancer*, **26**, 321.

LEWIS, W. H. & WEBSTER, L. T. (1921) Giant Cells in Cultures from Human Lymph Nodes. *J. exp. Med.*, **33**, 349.

LIEB, L. M. & LISCO, H. (1966) *In vitro* Uptake of Tritiated Thymidine by Carcinoma of the Human Colon. *Cancer Res.*, **26**, 733.

LITTLE, J. H. (1972) Histology and Prognosis in Cutaneous Malignant Melanoma. In *Melanoma and Skin Cancer*. Proc. Int. Cancer Conf. Ed. W. H. McCarthy, p. 107. Sydney.

LUKES, R. J. & BUTLER, J. J. (1966) The Pathology and Nomenclature of Hodgkin's Disease. *Cancer Res.*, **26**, 1063.

MACCARTY, W. C. (1922) Factors which Influence Longevity in Cancer. *Ann. Surg.*, **76**, 9.

MACCARTY, W. C. & MAHLE, A. E. (1921) Relation of Differentiation and Lymphocytic Infiltration to Postoperative Longevity in Gastric Carcinoma. *J. Lab. clin. Med.*, **6**, 473.

MARTIN, R. F. & BECKWITH, J. B. (1968) Lymphoid Infiltrates in Neuroblastomas: their Occurrence and Prognostic Significance. *J. pediat. Surg.*, **3**, 161.

MONAFO, W. W., KRAUSE, G. L. & MEDINA, J. G. (1962) Carcinoma of the Stomach: Morphological Characteristics Affecting Survival. *Archs Surg.*, **85**, 754.

MONIS, B. & WEINBERG, T. (1961) Cytochemical Study of Esterase Activity in Human Neoplasms and Stromal Macrophages. *Cancer, N.Y.*, **14**, 369.

MORRISON, A. S., BLACK, M. M., LOWE, C. R., MACMAHON, B. & YUASA, S. (1973) Some International Differences in Histology and Survival in Breast Cancer. *Int. J. Cancer*, **11**, 261.

MURPHY, J. B. (1926) The Lymphocyte in Resistance to Tissue Grafting, Malignant Disease, and Tuberculous Infection. *Monog. Rockefeller Inst. med. Res.*, No. 21.

NAIRN, R. C., NIND, A. P. P., GULI, E. P. G., MULLER, H. K., ROLLAND, J. M. & MINTY, C. C. J. (1971*a*) Specific Immune Response in Human Skin Carcinoma. *Br. med. J.*, iv, 701.

NAIRN, R. C., NIND, A. P. P., GULI, E. P. G., DAVIES, D. J., ROLLAND, J. M., MCGIVEN, A. R. & HUGHES, E. S. R. (1971*b*) Immunological Reactivity in Patients with Carcinoma of Colon. *Br. med. J.*, iv, 706.

NEWTON, K. A., MACKENZIE, D. H., SPITTLE, M. F. & MIKOLAJCZUK, A. (1973) Hodgkin's Disease—a Clinicopathological Study of 250 Cases with a 5-year Follow-up. *Br. J. Cancer*, **27**, 80.

NIND, A. P. P., NAIRN, R. C., ROLLAND, J. M., GULI, E. P. G. & HUGHES, E. S. R. (1973) Lymphocyte Anergy in Patients with Carcinoma. *Br. J. Cancer*, **28**, 108.

PAAVOLAINEN, M. (1970) Stroma Reactions as Prognostic Factors in Epidermoid Carcinoma of the Larynx. A Histological and Histochemical Study. *Diss. Helsinki.* (Cited by Paile, 1971).

PAILE, A. (1971) Morphology and Prognosis of Carcinoma of the Stomach. *Ann. chirurg. gynaec. Fenn.*, **60**, Suppl. 175.

PIERCE, G. B. (1966) Ultrastructure of Human Testicular Tumors. *Cancer, N.Y.*, **19**, 1963.

POWELL, L. D. (1923) The Relationship of Cellular Differentiation, Fibrosis, Hyalinization, and Lymphocytic Infiltration to Postoperative Longevity of Patients with Squamous Cell Epithelioma of the Skin and Lip. *Cancer Res.*, **7**, 371.

PREHN, R. T. (1972) The Immune Reaction as a Stimulator of Tumor Growth. *Science, N.Y.*, **176**, 170.

PRETLOW, T. G., LUBEROFF, D. E., HAMILTON, L. J., WEINBERGER, P. C., MADDOX, W. A. & DURANT, J. R. (1973) Pathogenesis of Hodgkin's Disease: Separation and Culture of Different Kinds of Cells from Hodgkin's Disease in a Sterile Isokinetic Gradient of Ficoll in Tissue Culture Medium. *Cancer, N.Y.*, **31**, 1120.

RICHTERS, A. & SHERWIN, R. P. (1965) Mitotic Lymphocytes in Primary Tissue Cultures of Normal and Neoplastic Human Lung. *Lab. Invest.*, **14**, 2122.

RICHTERS, A., SHERWIN, R. P. & RICHTERS, V. (1971) The Lymphocyte and Human Lung Cancers. *Cancer Res.*, **31**, 214.

ROBERTS, M. M., BASS, E. M., WALLACE, I. W. J. & STEVENSON, A. (1973) Local Immunoglobulin Production in Breast Cancer. *Br. J. Cancer*, **27**, 269.

ROSENTHAL, S. R. (1936) Significance of Tissue Lymphocytes in Prognosis of Lymphogranulomatosis. *Archs Path.*, **26**, 628.

SARMA, K. P. (1970) The Role of Lymphoid Reaction in Bladder Cancer. *J. Urol.*, **104**, 843.

SAXEN, E. & PENTTINEN, K. (1965) Differences in the Effect of Individual Human Sera on Cell Cultures. *J. natn. Cancer Inst.*, **35**, 67.

SCHOCH, E. O. (1926) Local Eosinophilia in Cancer. *Zentralbl. Gynäk.*, **50**, 2895 (abstract: (1927) *J. Am. med. Ass.*, **88**, 447).

SINKOVICS, J. G., SHIRATO, E., GYORKEY, J. R., CABINESS, J. R. & HOWE, C. D. (1970) Relationship between Lymphoid Neoplasms and Immunologic Function. In *Leukaemia-Lymphoma.* 14th Annual Clinical Conference, Univ. of Texas M.D. Anderson Hospital and Tumour Institute. Chicago. p. 53.

SISTRUNK, W. E. & MACCARTY, W. C. (1922) Life Expectancy Following Radical Amputation for Carcinoma of the Breast: a Clinical and Pathologic Study of 218 Cases. *Ann. Surg.*, **75**, 61.

STEWART, T. H. M. (1969) The Presence of Delayed Hypersensitivity Reactions in Patients towards Cellular Extracts of their Malignant Tumors. 2. A Correlation between the Histologic Picture of Lymphocytic Infiltration of the Tumour Stroma, the Presence of such a Reaction, and a Discussion of the Significance of this Phenomenon. *Cancer, N.Y.*, **23**, 1380.

STJERNSWÄRD, J., ALMGARD, L. E., FRANZEN, S., VON SCHREEB, T. & WADSTROM, L. B. (1970) Tumour-distinctive Cellular Immunity to Renal Carcinoma. *Clin. & exp. Immunol.*, **6**, 963.

TAKAHASHI, K. (1961) Squamous Cell Carcinoma of the Esophagus: Stromal Inflammatory Cell Infiltration as a Prognostic Factor. *Cancer, N.Y.*, **14**, 921.

TANAKA, T., COOPER, E. H. & ANDERSON, C. K. (1970) Lymphocyte Infiltration in Bladder Carcinoma. *Rev. Eur. Étud. clin. Biol.*, **15**, 1084.

TAYLOR, G. & ODILI, J. L. I. (1972) Histological Evidence of Tumour Rejection after Active Immunotherapy in Human Malignant Disease. *Br. med. J.*, ii, 183.

TUBIANA, M., ATTIE, E., FLAMANT, R., GERARD-MARCHANT, R. & HAYAT, M. (1971) Prognostic Factors in 454 Cases of Hodgkin's Disease. *Cancer Res.*, **31**, 1801.

UNDERWOOD, J. C. E. (1973) *The Ultrastructure and Interactions of Lymphoreticular Cells in Human Neoplasms.* M.D. Thesis. University of London.

UNDERWOOD, J. C. E. & CARR, I. (1972) The Ultrastructure of the Lymphoreticular Cells in Non-lymphoid Human Neoplasms. *Virchows Arch. path. Anat.*, Abt. B, **12**, 39.

VIRCHOW, R. (1863) *Krankhaften Geschwülste.* Berlin.

WADE, H. (1908) An Experimental Investigation of Infective Sarcoma in the Dog, with a Consideration of its Relationship to Cancer. *J. Path. Bact.*, **12**, 384.

WALDEYER, H. W. G. (1872) Die entwicklung der carcinome. *Virchows Arch. path. Anat.*, **55**, 67.

WASSERMAN, S. I., GOETZL, E. J., ELLMAN, L. & AUSTEN, F. K. (1974) Tumor-associated Eosinophilotactic Factor. *N. Engl. J. Med.*, **290**, 420.

WHITE, W. C. (1927) Late Results of Operation for Carcinoma of the Breast. *Ann. Surg.*, **86**, 695.

WIENER, J. (1970) Ultrastructural Aspects of Delayed Hypersensitivity. *Curr. top. Path.*, **52**, 143.

YOON, I. L. (1959) The Eosinophil and Gastrointestinal Carcinoma. *Am. J. Surg.*, **97**, 195.